# 4438 Twenty-four-hour Urinary Sodium Excretion Estimated from a Spot Urine Sample May Be Used as an Indicator of Intake in CKD Patients

**DOI:** 10.1017/cts.2020.155

**Published:** 2020-07-29

**Authors:** Andrea Lobene, Elizabeth Stremke, Ranjani Moorthi, Sharon Moe, Kathleen M Hill Gallant

**Affiliations:** 1 Indiana University School of Medicine; 2 Purdue University

## Abstract

OBJECTIVES/GOALS: Sodium (Na) intake can elevate blood pressure and is a factor in developing chronic kidney disease (CKD). Twenty-four-hour urinary Na (24hUNa) is the gold standard for assessing Na intake but is burdensome. Validated equations estimate 24hUNa (e24hUNa) from a spot urine sample, but these estimations are not validated against a known Na intake in CKD. METHODS/STUDY POPULATION: The current study is a secondary analysis of a 9-day controlled feeding study in moderate CKD patients matched to healthy adults. Only CKD patients were used for the current analyses (n = 8). Participants consumed a controlled diet for 9 days, providing ~2400 mg Na/d as determined by inductively coupled plasma optical emission spectroscopy (ICP). On days 7 and 8, participants collected all urine in an inpatient setting, beginning with a fasting sample on day 7. Urine sample mineral analyses were performed by ICP and urinary creatinine by the Jaffe reaction. The day 7 fasting urine sample was used to calculate e24hUNa using 6 published equations. Log-transformed Na intake, measured 24hUNa, and e24hUNa were compared by repeated-measures ANOVA with planned contrasts using SAS. RESULTS/ANTICIPATED RESULTS: Fifty percent of the CKD patients (n = 4) were female; 63% (n = 5) were white, and 37% (n = 3) were black. On average, participants were aged 56.6 ± 13.8 y with a BMI of 31.7 ± 9.4 kg/m^2^ and eGFR of 40.7 ± 7.9 mL/min. Based on actual food intake, average Na intake on day 7 was 2024 ± 388 mg. Average measured 24hUNa was 2529 ± 1334 mg. The main ANOVA was significant (p = 0.02). Results from the planned contrasts found that e24hUNa from the SALTED cohort, an equation developed specifically for CKD patients, was significantly higher than both Na intake (p<0.001) and measured 24hUNa (p = 0.007). For the remaining 5 equations, e24hUNa was not significantly different from measured 24hUNa nor dietary Na intake. DISCUSSION/SIGNIFICANCE OF IMPACT : Our results suggest that e24hUNa calculated using most published equations may provide a reliable and low-burden method of assessing dietary Na intake in moderate CKD patients. These findings should be confirmed in larger samples. Additional studies are needed to validate or dispute the use of the SALTED equation for estimating Na intake.

